# Type I Interferonopathy due to a Homozygous Loss-of-Inhibitory Function Mutation in STAT2

**DOI:** 10.1007/s10875-023-01445-3

**Published:** 2023-02-08

**Authors:** Gaofeng Zhu, Mihaly Badonyi, Lina Franklin, Luis Seabra, Gillian I. Rice, Anne-Boland-Auge, Jean-François Deleuze, Salima El-Chehadeh, Mathieu Anheim, Anne de Saint-Martin, Sandra Pellegrini, Joseph A. Marsh, Yanick J. Crow, Marie-Therese El-Daher

**Affiliations:** 1 MRC Human Genetics Unit, Institute of Genetics and Cancer, The University of Edinburgh, Edinburgh, UK; 2 Cytokine Signalling Unit, Institut Pasteur, Paris, France; 3 Institut Imagine, Paris, France; 4 Division of Evolution, Infection and Genomics, The University of Manchester, Manchester, UK; 5 Centre National de Recherche en Génomique Humaine (CNRGH), Université Paris-Saclay, CEA, Evry, France; 6 Institut de Génétique Médicale d’Alsace, Strasbourg, France; 7 Service de Neurologie, Centre de Référence Des Maladies Neurogénétiques Rares, Hôpitaux Universitaires de Strasbourg, Strasbourg, France; 8 Fédération de Médecine Translationnelle de Médecine de Strasbourg, Strasbourg, France; 9 Institut de Génétique Et de Biologie Moléculaire Et Cellulaire, UMR7104, INSERM-U964/CNRS, Université de Strasbourg, Illkirch, France; 10 Unité de Neurologie Pédiatrique, Centre de Référence Des Epilepsies Rares, Hôpitaux Universitaires de Strasbourg, Strasbourg, France; 11 UMR 7104 INSERM U1258, IGBMC-CNRS, Strasbourg, France

**Keywords:** Type I interferonopathy, Interferon stimulated genes, STAT2, USP18

## Abstract

**Purpose:**

STAT2 is both an effector and negative regulator of type I interferon (IFN-I) signalling. We describe the characterization of a novel homozygous missense STAT2 substitution in a patient with a type I interferonopathy.

**Methods:**

Whole-genome sequencing (WGS) was used to identify the genetic basis of disease in a patient with features of enhanced IFN-I signalling. After stable lentiviral reconstitution of STAT2-null human fibrosarcoma U6A cells with STAT2 wild type or p.(A219V), we performed quantitative polymerase chain reaction, western blotting, immunofluorescence, and co-immunoprecipitation to functionally characterize the p.(A219V) variant.

**Results:**

WGS identified a rare homozygous single nucleotide transition in *STAT2* (c.656C > T), resulting in a p.(A219V) substitution, in a patient displaying developmental delay, intracranial calcification, and up-regulation of interferon-stimulated gene (ISG) expression in blood. In vitro studies revealed that the STAT2 p.(A219V) variant retained the ability to transduce an IFN-I stimulus. Notably, STAT2 p.(A219V) failed to support receptor desensitization, resulting in sustained STAT2 phosphorylation and ISG up-regulation. Mechanistically, STAT2 p.(A219V) showed defective binding to ubiquitin specific protease 18 (USP18), providing a possible explanation for the chronic IFN-I pathway activation seen in the patient.

**Conclusion:**

Our data indicate an impaired negative regulatory role of STAT2 p.(A219V) in IFN-I signalling and that mutations in STAT2 resulting in a type I interferonopathy state are not limited to the previously reported R148 residue. Indeed, structural modelling highlights at least 3 further residues critical to mediating a STAT2-USP18 interaction, in which mutations might be expected to result in defective negative feedback regulation of IFN-I signalling.

**Supplementary Information:**

The online version contains supplementary material available at 10.1007/s10875-023-01445-3.

## Introduction

Type I interferon (IFN-I) signalling drives a complex downstream transcriptional network crucial to host defense against invading pathogens [1]. Almost all cells in the human body can express some amount of IFN-I upon appropriate stimulation [2]. After production, type I IFNs are secreted and, in an autocrine or paracrine manner, bind to IFN-I receptors (IFNARs). IFNARs consist of two subunits, namely, IFNAR1 and IFNAR2, which are phosphorylated upon ligand binding, leading to the activation of the receptor-associated Janus kinase (JAK) family members tyrosine kinase 2 (TYK2) and JAK1, respectively [3]. In turn, TYK2 and JAK1 recruit and activate signal transducer and activator of transcription 1 (STAT1) and STAT2 through phosphorylation. Phosphorylated STAT1 and STAT2, together with IFN regulatory factor 9 (IRF9), form a complex, named IFN-stimulated gene factor 3 (ISGF3), which translocates into the nucleus and acts as a transcriptional activator by binding to IFN-sensitive response elements (ISREs) within a broad repertoire of so-called IFN-stimulated genes (ISGs). ISG proteins play diverse roles in modifying the innate and adaptive immune systems, restricting pathogen survival and growth, and regulating cell proliferation, survival and death.

IFN-I production and signalling require tight regulation, with a failure of such regulation having severe consequences. As examples, *IFNAR2* deficiency results in potentially fatal MMR (measles, mumps, and rubella) vaccination-related encephalitis [4], while deficiency of *USP18*, a known negative regulator of IFN-I signalling, is associated with a type I interferonopathy state, where patients can exhibit congenital microcephaly, thrombocytopenia, hepatic dysfunction, and hepatosplenomegaly [5]. In mice, Usp18 negatively regulates Stat1 activation and the downstream IFN-I response by interaction with Ifnar2, and mice lacking *Usp18* in microglia display brain disease due to uncontrolled IFN-I signalling [6].

Here, in a patient with clinical features of a type I interferonopathy, we describe the identification of a homozygous single nucleotide transition in *STAT2* (c.656C > T) which results in an alanine 219 to valine 219 substitution (p.(A219V)) in STAT2. A role for STAT2 as an effector of IFN-I signalling was reported more than 30 years ago (summarized in reference [7]). In 2017, another role of STAT2 was uncovered by Arimoto et al. [8]: specifically, in the later stage of IFN-I signalling, STAT2 was shown to bind to USP18, leading to a displacement of phosphorylated JAK1 from IFNAR2 and a shutdown of the IFN-I induced signalling cascade. Duncan et al. subsequently described two patients from the same family with a homozygous *STAT2* mutation specifically affecting this negative feedback regulatory role of STAT2 [9]. In their study, STAT2 p.(R148W) lost the ability to bind USP18, so that IFN-I signalling was abnormally activated. These patients demonstrated intracranial calcification, systemic inflammation, and multiorgan dysfunction. Shortly thereafter, Gruber et al. identified another patient with a mutation involving the same amino acid residue of STAT2 [10]. In this case, the mutation, p.(R148Q), retained USP18-binding capacity, but the STAT2-USP18 dimer could not traffic to IFNAR2 (so as to displace JAK1), also resulting in enhanced IFN-I signalling. This patient shared some of the same clinical features observed in the two patients described by Duncan et al.

## Methods

### Whole-Genome Sequencing (WGS)

WGS was performed by the Commissariat à l'énergie atomique et aux énergies alternatives (CEA), as part of a collaboration between CEA-IBFJ/CNRGH, Institut Imagine, INSERM and Université Paris Descartes. One microgram of genomic DNA was used to prepare a library for WGS using the Illumina TruSeq DNA PCR-free library preparation kit, according to the manufacturer’s instructions. After normalization and quality control, qualified libraries were sequenced on a HiSeq X Five platform (Illumina), as paired-end 150 base pair reads. One lane of the HiSeq X Five flow cell was used for each sample to reach an average sequencing depth of 30 × . The sequence quality parameters were assessed throughout the sequencing run, and standard bioinformatics analysis of sequencing data was based on the Illumina pipeline to generate FASTQ files for each sample. Variants were filtered according to a frequency on gnomAD of < 0.0001 and < 10 occurrences in our in-house variant database.

### Cells and Cytokine

STAT2-deficient human fibrosarcoma cell line U6A and human embryonic kidney (HEK) 293FT cells were both cultured in Dulbecco’s modified Eagle’s medium (DMEM) supplemented by 10% fetal calf serum (FCS) and 1% penicillin/streptomycin (p/s). Unless otherwise specified, human recombinant IFNα2b (11,105–1, PBL Assay Science) was used at 250 IU/mL.

### Interferon Signature Testing

The analysis of 24 genes and 3 housekeeping genes was conducted using the NanoString customer designed CodeSets according to the manufacturer’s recommendations (NanoString Technologies, Seattle, WA). One hundred nanograms of total RNA was loaded for each sample. Agilent Tapestation was used to assess the quality of the RNA. Data were processed with nSolver software (NanoString Technologies Seattle, WA). The data were normalized relative to the internal positive and negative calibrators, the three reference probes, and healthy control samples. The median of the 24 probes for each of 29 healthy control samples was calculated. The mean NanoString score of 29 healthy controls + 2 SD of the mean was calculated. Scores above this value (2.75) were designated as positive. Probes were *IFI27*, *IFI44L*, *IFIT1*, *ISG15*, *RSAD2*, *SIGLEC1*, *CMPK2*, *DDX60*, *EPSTI1*, *FBXO39*, *HERC5*, *HES4*, *IFI44*, *IFI6*, *IFIH1*, *IRF7*, *LAMP3*, *LY6E*, *MX1*, *NRIR*, *OAS1*, *OASL*, *OTOF*, and *SPATS2L*. Reference probes were *HPRT1*, *NRDC*, and *OTUD5*. Generation of an interferon signature by qPCR of a 6-gene panel is as described in Rice et al. [11] (and see the “RNA isolation and qPCR” section below for further details of the probes used).

### Lentiviral Constructs, Site-Directed Mutagenesis, and Lentiviral Transduction

The bicistronic lentivirus vector (pHR-SIN-CSGW) containing the full length of WT human *STAT2* or p.(R148W), as well as pLenti6/V5 vector containing the full length of WT human *USP18*, was a kind gift from Dr. Christopher Duncan (Newcastle University, UK). Site-directed mutagenesis on pHR-SIN-CSGW-STAT2 WT to generate p.(A219V) mutant was carried out with Q5 site-directed mutagenesis kit (E0552S, New England BioLabs) according to the manufacturer’s instructions and verified by Sanger sequencing. Primer sequences for mutagenesis were GCCTCCAAAGTACTGCTAGGC (forward) and ATCCAGCACCTCCTTTCTC (reverse).

Lentiviruses were produced by co-transfection of pCMV-VSV-G (Addgene plasmid no. 8454), psPAX2 (Addgene plasmid no. 12260), and lentiviral transfer plasmid (pHR-SIN-CSGW-STAT2 WT, pHR-SIN-CSGW-STAT2 p.(A219V), or pHR-SIN-CSGW-STAT2 p.(R148W)) in HEK 293FT cells using Lipofectamine 2000 Transfection Reagent (11,668,019, Invitrogen,). Twenty-four-hour post-transfection, cells were refreshed with DMEM supplemented with 10% FCS and 1% p/s. Forty-eight-hour post-transfection, virus-containing supernatant was collected and filtered through 0.45-μm sterile filter. U6A cells were then incubated with virus-containing supernatant supplemented with 8 μg/mL polybrene (TR-1003-G, Merk Millipore) for 24 h, and cells were then refreshed with DMEM supplemented with 10% FCS and 1% p/s. Forty-eight-hour post-transduction, cells were subjected to selection with puromycin (2.0 μg/mL) (A11138-03, Gibco).

### RNA Isolation and qPCR

U6A cells were lysed in TRIzol reagent (15,596,026, Thermo Fisher Scientific), and RNA was subsequently extracted with Direct-zol RNA MiniPrep kit (R2050, Zymo Research) following the manufacturer’s instructions. RNA from whole-blood samples collected in PAXgene tubes (672,165, PreAnalyticX) was extracted using PAXgene blood RNA kit (762,174, PreAnalyticX). RNA was reverse-transcribed using high-capacity cDNA reverse transcription kit (4,368,814, Thermo Fisher Scientific). The expression of ISGs (*MX1*, *ISG15*, *USP18*, *IFI27*, *IFIT1*, *IFI44L*, *SIGLEC*, and *RSAD2*), relative to the *BACT* and *GAPDH* housekeeping genes, was analyzed by TaqMan quantitative real-time PCR (TaqMan Fast Universal PCR Master Mix (2 ×) No AmpErase UNG, 4,352,042, Thermo Fisher Scientific) on a 7900HT Sequence Detection System (Applied Biosystems). The TaqMan probes were Hs00895608_m1 (*MX1*), Hs00192713_m1 (*ISG15*), Hs00276441_m1 (*USP18*), Hs01086370_m1 (*IFI27*), Hs00356631_g1 (*IFIT1*), Hs00199115_m1 (*IFI44L*), Hs00988061_g1 (*SIGLEC1*), Hs01057264_m1 (*RSAD2*), Hs01060665_g1 (*BACT*), and Hs02786624_g1 (*GAPDH*). The relative levels of ISG transcription were calculated by the ∆∆Ct method, relative to the mean values for the mock-treated controls or healthy donors.

### Immunoblotting

Whole-cell lysates for immunoblotting were prepared by incubating cells for 1 h at 4 °C with rotation in lysis buffer (25 mM Tris–HCl, pH 8.0, 1% NP-40, 150 mM NaCl, 1.5 mM MgCl2, 0.05% SDS, 0.5% sodium deoxycholate, supplemented with protease inhibitor cocktail (04,693,159,001, Roche)). Samples were then centrifuged at 12,000 rpm at 4 °C for 10 min. Supernatant containing soluble protein fraction was collected, and protein concentration was measured with Pierce BCA protein assay kit (23,227, Thermo Fisher Scientific).

Thirty micrograms of protein from each sample with Pierce Lane marker reducing sample buffer (39,000, Thermo Fisher Scientific) was denatured at 95 °C for 10 min and resolved on NuPage 4–12% Bis–Tris Gels (NP0336BOX, Invitrogen) in NuPage MOPS SDS running buffer (NP0001, Invitrogen). Proteins were then transferred onto the nitrocellulose membrane of an iBlot 2 NC Regular Stack (IB23001, Invitrogen) for 15 min at 15 V using the iBlot 2 Dry Blotting System (IB21001, Invitrogen). Membranes were blocked in intercept (TBS) blocking buffer (927–60,001, LI-COR) for 30 min at room temperature and incubated overnight at 4 °C with primary antibodies of interest in blocking solution supplemented with 0.1% Tween 20 (EC-607, National Diagnostics). Primary antibodies used were STAT2 (sc-1668, Santa Cruz Biotechnology), p-STAT2 (07–224, Merk/Millipore), STAT1 (9176, Cell Signaling Technology), p-STAT1 (7649, Cell Signaling Technology), MX1 (ab95926, abcam), ISG15 (NBP1-04,310, Novus Biologicals), USP18 (4813 s, Cell Signaling Technology), Cofilin (5175 s, Cell Signaling Technology), and Vinculin (13,901, Cell Signaling Technology). IRdye-conjugated anti-mouse (926–68,070, LI-COR) or anti-rabbit (925–32,211, LI-COR) secondary antibodies diluted in intercept (TBS) blocking buffer plus TBS supplemented with 0.1% Tween 20 (TBS-T) (intercept (TBS) blocking buffer: TBS-T = 1:2 v/v) were used to detect targeted proteins. Membranes were scanned using the Odyssey CLx System (LI-COR). Densitometry quantification and analyses were performed using the Image Studio Lite software v.5.2 (LI-COR).

### Co-Immunoprecipitation (co-IP)

HEK 293FT cells were transiently transfected with pLenti6/V5-USP18 together with either pHR-SIN-CSGW-STAT2 WT or pHR-SIN-CSGW-STAT2 p.(A219V) using Lipofectamine 2000 Transfection Reagent and Opti-MEM Reduced Serum Medium, GlutaMAX Supplement (51,985,034, Gibco). Six-hour post-transfection, cells were refreshed with DMEM supplemented with 10% FCS and 1% p/s. Twenty-four-hour post-transfection, cells were lysed in IP buffer (50 mM Tris–HCl, pH 7.5, 0.5% NP-40, 200 mM NaCl, 10% glycerol, 1 mM EDTA) supplemented with protease inhibitor cocktail (04,693,159,001, Roche). Lysates were collected by centrifugation at 12,000 rpm at 4 °C for 10 min, and soluble fractions are collected to measure protein concentration with Pierce BCA protein assay kit. Lysates with the same amount of protein for each sample were then incubated with anti-STAT2 (A-7) antibody (sc-1668, Santa Cruz Biotechnology) overnight at 4 °C with rotation. Lysates were then incubated with Dynabeads Protein G (10003D, Invitrogen) for 2 h at 4 °C with rotation. Immunoprecipitates were eluted with Pierce Lane marker reducing sample buffer before being subjected to immunoblotting as previously described.

### Immunofluorescence

Stably transduced U6A cells (with either pHR-SIN-CSGW-STAT2 WT or pHR-SIN-CSGW-STAT2 p.(A219V)) grown on coverslips were fixed with 4% paraformaldehyde in PBS for 15 min at room temperature, before being permeabilized with 0.1% Triton X-100 (T9284, Sigma-Aldrich) in PBS and then blocked in 1% normal goat serum in PBS. Cells were incubated for 1 h at room temperature with anti-STAT2 (A-7) primary antibody (1:100 v/v in PBS; sc-1668, Santa Cruz Biotechnology) and then washed 3 times with PBS. Secondary antibody incubation was performed with goat anti-mouse Alexa Fluor 594 (4 μg/mL; A11032, Thermo Fisher Scientific) for 1 h at room temperature in the dark followed by 3 times of PBS wash. Nuclear staining was then performed with 4′,6-diamidino-2-phenylindole (DAPI; 1 μg/mL; Thermo Fisher Scientific) for 5 min at room temperature in the dark followed by 3 times of PBS wash. Coverslips were mounted on glass slides with ProLong Gold anti-fade reagent (P36934, Invitrogen). Cells were imaged with a Stellaris confocal microscope with a 63 × oil immersion objective (Leica). STAT2-deficient cells were used to demonstrate the specificity and lack of non-specific background for this staining method. Image analysis was performed with ImageJ.

### Structure Modelling of STAT2-USP18 Interaction

Deletion mutagenesis in combination with coimmunoprecipitation assays has previously identified the amino acid region 138–572 of STAT2 and 51–112 of USP18 to be important for their interaction [8]. More recently, arginine 148 in STAT2 has been suggested to be directly involved in USP18 binding [9]. Based upon these criteria, molecular docking was performed to obtain a potential model of the interaction. The structure of STAT2 was obtained from the AlphaFold database removing residues 709–851 of the highly disordered C terminus. Then, a homology model of USP18 was generated with Phyre2 [12], using the available experimental structures in the Protein Data Bank [13]. ClusPro was adopted [14] to dock the two proteins by deriving loose restraints in the following manner: leucine 103 was deemed to be the residue closest to the centroid of the region 51–112 in the structure of USP18. A 20 Å restraint between arginine 148 (STAT2) and leucine 103 (USP18) was set to allow a large rotational space to be explored by decoys during docking covering the entire 51–112 region of USP18. The final model was taken as the top-ranking model of the hydrophobic-favored scoring scheme [14].

### *In Silico* Deep Mutagenesis of the Predicted STAT2-USP18 Interface

To prioritize substitutions in STAT2 that are likely to disrupt the interaction with USP18, we first determined the interface residues based upon the difference in the solvent accessible surface of STAT2 as a monomer and in complex with USP18 using FreeSASA 2.0.3 [15]. Then, the structural effect of each of 19 substitutions of interface residues was calculated, measured as the predicted Gibbs free energy change (ΔΔ*G*) by FoldX 5.0 [16]. The “RepairPDB” command was run first to minimize the structures, and the ΔΔ*G* values in the monomer (ΔΔ*G*_monomer_) and in the complex (ΔΔ*G*_full_) were calculated with the “BuildModel” command as the average of ten replicates. A rule-based method was applied to rank the substitutions. First, variants observed in gnomAD were excluded [17]. Second, only mutations with ΔΔ*G*_monomer_ values between − 0.5 and + 0.5 were considered, which would more likely allow folding of the protein (mean ΔΔ*G*_monomer_ of 14 gnomAD variants at the interface is 0.309). Third, mutations with ΔΔ*G*_full_ > 2, were prioritized, i.e., those that are likely to have a disrupting effect at the interface (only 3 out of 14 gnomAD variants are above this value with the maximum of 3.23). Lastly, the subset of mutations to residues that are no more than 5 Å distance away from the nearest atoms of residues R148 and A219 were further restricted to maximize the chance of the residue being biologically and thus pathologically important. Through this procedure, 10 substitutions from the possible 456 substitutions of 24 interface residues are shortlisted, with the 3 affected residues involved in these 10 substitutions highlighted in Fig. S3.

### Statistical Analysis

The number of experiments and the statistical tests performed are indicated in the figure legends. Statistical testing was undertaken in GraphPad Prism 9. Error bars represent standard error of the mean (SEM).

## Results

### Severe Neurological Disease and Systemic Inflammation Associated with Excessive IFN-I Signalling in Blood

**Fig. 1:**
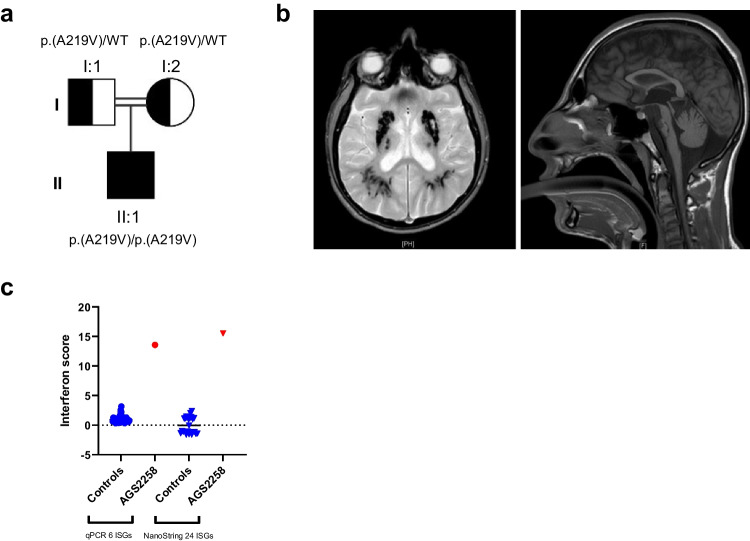
HYPERLINK "sps:id::fig1||locator::gr1||MediaObject::0" Identification of a patient (AGS2258) presenting with neurological abnormalities and enhanced interferon (IFN) signalling in blood. **a** Pedigree. Filled symbol indicates the affected patient, half-filled symbols indicate unaffected heterozygous parents, squares indicate males, circle indicates female, and the double line indicates consanguinity. WT, wild type. p.(A219V) and WT refer to STAT2 genotypes. **b** Neuroimaging showing intracranial calcification (dark signal voids) (left: axial GE) and microcephaly, small pons and cerebellar hemispheres (right: sagittal T1). **c** qPCR and NanoString analyses of IFN-stimulated genes (ISGs) in whole blood RNA isolated from the patient and healthy controls

We evaluated a male patient (AGS2258) born to first cousin consanguineous Turkish parents (Fig. 1a). Briefly, he presented with fever and deranged liver function at age 5 months. Cranial MRI and CT at age 8 months revealed intracranial calcification and cerebral atrophy (Fig. 1b). He began to walk at 18 months of age, and talk at age 3 years. Subsequently, he was recognized to demonstrate a progressive spastic paraparesis. Several stroke-like episodes of acute hemiparesis occurred between 2 and 3 years of age, sometimes apparently associated temporally with fever in the context of viral infection. Later, at age 3.5 years, he experienced a pontine hemorrhage, followed by a pseudobulbar palsy and spastic dystonic tetraparesis. His head circumference at age 19 years was 49.5 cm (− 5 SD), height 150 cm (− 4 SD), and weight 40 kg (< < 1 centile). The patient is currently alive at age 23 years, with very little language ability although he can still communicate. He demonstrates a spastic dystonia and is able to walk a few steps with help but normally uses a wheelchair. The patient has not experienced any unusual susceptibility to infection, has received a full program of vaccinations (Pentavalent vaccine, MMR, BCG) without untoward effect, and does not currently manifest any overt inflammatory features. He is on no regular medication beyond trihexyphenidyl and baclofen for his neurological dysfunction.

This clinical phenotype is consistent with a severe type I interferonopathy. In line with this, at age 18 years, we measured whole-blood mRNA levels of 6 ISGs [11] by quantitative polymerase chain reaction (qPCR) (Fig. 1c). Compared to the composite data derived from 29 healthy donor controls, the expression of all 6 ISGs was elevated in the patient. Further, NanoString analysis, a technique to measure individual RNA molecules, assessed at age 20 years, showed a similar up-regulation of the expression of a larger panel of 24 ISGs (Fig. 1c), indicating a persistent IFN-mediated inflammatory state.

### Homozygous Missense Substitution in STAT2 Identified Through Whole-Genome Sequencing (WGS)

**Fig. 2:**
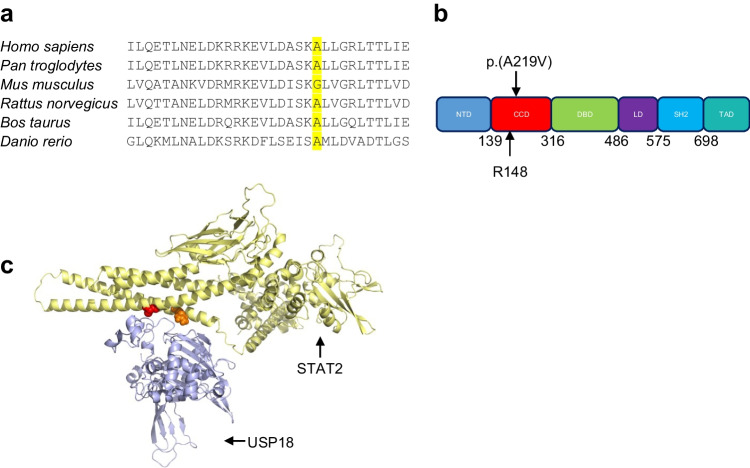
STAT2 protein. **a** STAT2 A219 is conserved across species, except in mouse. **b** Cartoon of the protein structure of STAT2. The p.(A219V) substitution identified in AGS2258 and the R148 amino acid residue mutated in the patients described by Duncan et al. and Gruber et al. are indicated. NTD, N-terminal domain; CCD, coiled-coil domain; DBD, DNA-binding domain; LD, linker domain; SH2, Src homology 2 domain; TAD, trans-activation domain. **c** Structural model showing interaction of STAT2 (yellow) and USP18 (pale blue). Alanine 219 is depicted in red and arginine 148 in orange

Taking a candidate gene approach to the WGS data, and concentrating on homozygous variants given the recorded parental consanguinity, we noted a very rare *STAT2* variant in the homozygous state in our patient, where a single nucleotide transition c.656C > T (transcript: NM_005419.4) results in an alanine to valine substitution at residue 219. The patient’s asymptomatic parents were heterozygous for this variant, which is present only once on gnomAD and is not present in the Greater Middle East Variome database. In silico analysis using SIFT and Polyphen predicted this STAT2 p.(A219V) substitution as “Deleterious” and “Possibly damaging” (score 0.698), with a CADD score of 17.81. STAT2 protein sequence alignment across different species showed that the A219 residue is well conserved (Fig. 2a). The affected amino acid residue A219 is located in the coiled-coil domain (CCD) of STAT2 (Fig. 2b), which is essential for USP18 binding and thus restricting excessive IFN-I signalling [8]. Notably, the two previously reported homozygous STAT2 mutations affect the same R148 residue that is also in this domain [9, 10]. 3D modelling of the STAT2-USP18 heterodimer showed both the amino acid residues, A219 and R148, to localize to the interface between STAT2 and USP18 (Fig. 2c), suggesting that the A219, like the R148, residue might play an essential role in mediating a STAT2-USP18 protein interaction.

### STAT2 p.(A219V) Leads to Prolonged Activation and Elevated ISG Expression upon IFNα2b Stimulation *In Vitro*

**Fig. 3:**
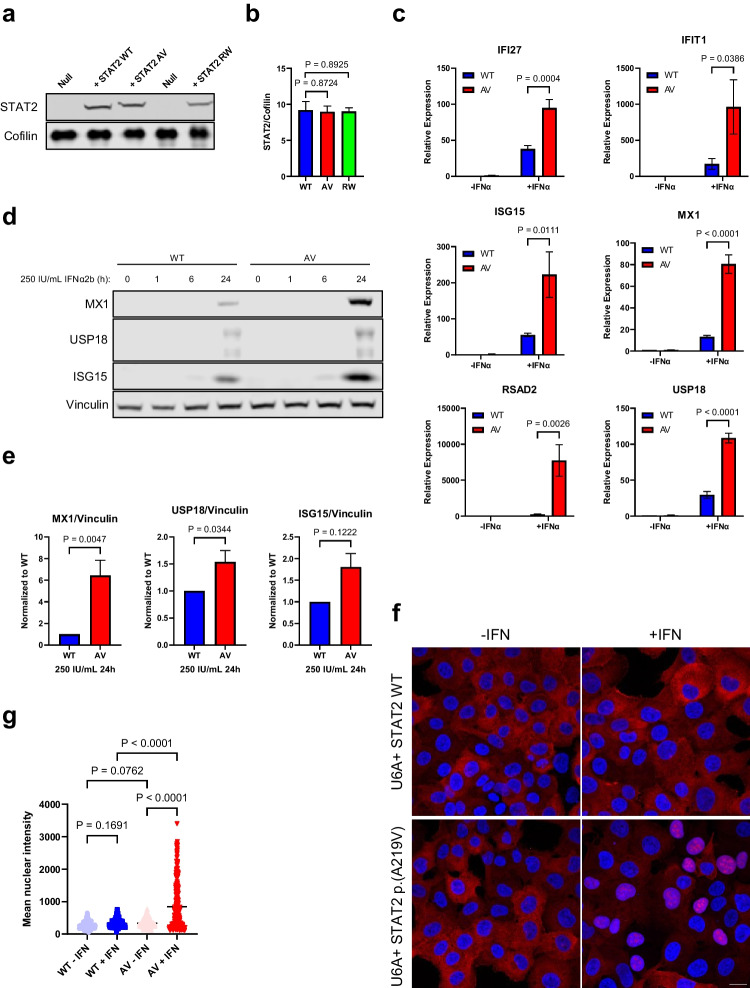
Cells transduced with the STAT2 p.(A219V) substitution display heightened sensitivity to IFN stimulation. **a** Immunoblotting analysis of U6A cells stably transduced with STAT2 WT, p.(A219V) (“AV”) or p.(R148W) (“RW”). **b** Densitometry quantification as in **a**. *n* = 3; unpaired *t* test. **c** qPCR analysis of ISG transcription in U6A cells stably transduced with either STAT2 WT or p.(A219V) and stimulated for 16 h with 250 IU/mL IFNα2b. *n* = 3; two-way ANOVA with Sidak’s multiple comparisons test. **d** ISG expression in time course following 250 IU/mL IFNα2b stimulation. **e** Densitometry quantification as in **d**. MX1, *n* = 4; USP18, *n* = 4; ISG15, *n* = 2; unpaired *t* test. **f** Immunofluorescence analysis of cells transduced with STAT2 WT or p.(A219V) and stimulated with 250 IU/m IFNα2b for 24 h. Blue, DAPI; red, STAT2. Scale bar represents 20 micrometers **g** Fluorescence signal intensity quantification as in **f**. *n* = 2; a total of 346 cells were quantified; two-way ANOVA with Sidak’s multiple comparisons test

In order to functionally characterize the cellular and molecular consequences of the p.(A219V) substitution, and in the absence of patient material, particularly dermal fibroblasts, we performed stable lentiviral transduction of STAT2-deficient U6A human fibrosarcoma cells [18, 19] with STAT2 WT and mutant (p.(A219V); p.(R148W)) constructs. Assessing the STAT2 protein level in transduced cells by western blot, all 3 transduced clones demonstrated stable STAT2 expression, with no significant difference between WT and p.(A219V) and WT and p.(R148W) (Fig. 3a, b).

Next, we performed stimulation experiments with different concentrations of IFNα2b for 30 min in cells transduced with STAT2 WT or p.(A219V). At a concentration of 250 IU/mL, STAT2 p.(A219V) showed higher phosphorylation levels compared to WT (Fig. S1a, b), indicating that the p.(A219V) mutant protein was able to transduce an IFN-I stimulus, and which was enhanced. We then performed qPCR analysis in transduced cells stimulated with 250 IU/mL IFNα2b for 16 h (Fig. 3c; Fig. S2a). It is notable that at baseline, i.e., without IFNα2b stimulation, no difference in ISG transcripts was observed between cells transduced with STAT2 WT, p.(A219V) (Fig. 3c) or the previously described mutant p.(R148W) (Fig. S2a). However, compared to cells transduced with STAT2 WT, cells transduced with p.(A219V) displayed elevated levels of *IFI27*, *IFIT1*, *ISG15*, *MX1*, *RSAD2*, and *USP18* transcripts after IFNα2b stimulation (Fig. 3c). Similarly, in agreement with the findings of Duncan et al. [9], cells transduced with p.(R148W) showed elevated ISG transcript expression after IFNα2b stimulation (Fig. S2a).

Next, we carried out a time-course experiment following stimulation with 250 IU/mL IFNα2b in WT and p.(A219V) cells (Fig. 3d). In line with the qPCR results, in western blot analysis, at the 24-h time point, p.(A219V) cells expressed higher amounts of MX1, USP18, and ISG15 proteins compared to STAT2 WT cells (Fig. 3e).

ISG transcription and expression are driven by the nuclear translocation of ISGF3, a hetero-trimer complex composed of p-STAT1, p-STAT2, and IRF9. To further assess if enhanced ISG expression in p.(A219V) cells was caused by prolonged STAT2 activation, we performed immunofluorescence in WT and p.(A219V) cells stimulated with 250 IU/mL IFNα2b for 24 h (Fig. 3f–g). In these experiments, STAT2 WT cells showed a STAT2 cytoplasmic staining. In contrast, cells with STAT2 p.(A219V) showed both cytoplasmic and nuclear STAT2 staining, suggesting prolonged IFNα2b signalling activation.

In summary, the above data indicate that the p.(A219V) mutation is able to transduce an IFN-I stimulus, of which the response is heightened and prolonged compared to STAT2 WT.

### STAT2 p.(A219V) Fails to Induce IFNAR2-Mediated Negative Feedback due to Defective USP18 Binding

**Fig. 4:**
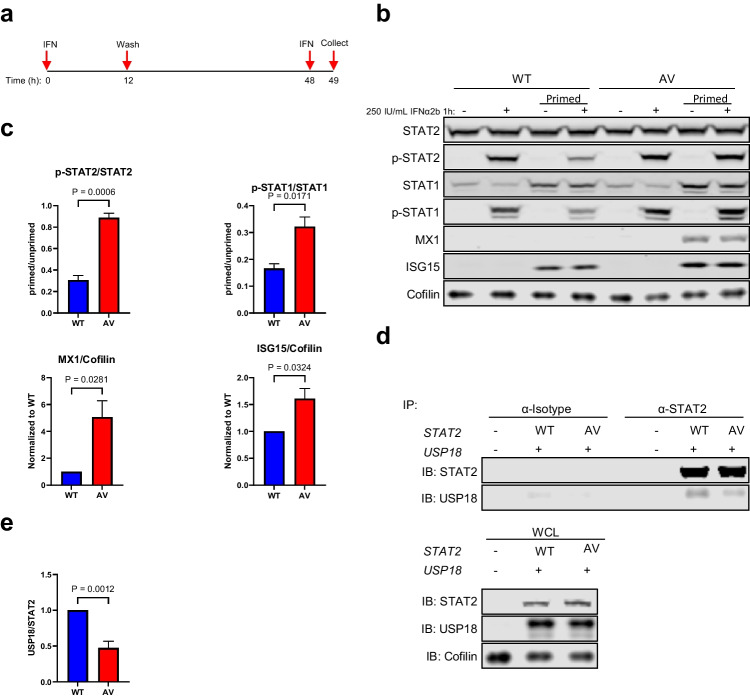
Cells transduced with the p.(A219V) substitution lose the capacity to restrict IFN signalling upon a second stimulation due to defective binding with USP18. **a** Experimental design. **b** Immunoblotting analysis of negative regulation function of STAT2 p.(A219V) (“AV”) by stimulating cells with (‘‘primed’’), or without, a priming stimulus. **c** Densitometry quantification as in **b**. *n* = 3; unpaired *t* test. **d** Co-immunoprecipitation of USP18 by STAT2 in HEK 293FT cells transiently transfected with STAT2 WT or p.(A219V), together with USP18*.* **e** Densitometry quantification as in **d**. USP18/STAT2, ratio to WT; *n* = 4; unpaired *t* test

STAT2 p.(A219V) retains the ability to transduce IFN-I signalling, associated with a prolonged and enhanced response to an IFN-I stimulus compared to STAT2 WT. We hypothesized the cause of this disturbance to be an impairment of the role of STAT2 p.(A219V) in negative feedback signalling. To explore this possibility, a priming experiment was designed (Fig. 4a). Here, we first primed WT and p.(A219V) cells with 250 IU/mL IFNα2b for 12 h, washed them extensively, and let the cells rest for 36 h. Then, we re-stimulated the cells with a second dose of 250 IU/mL IFNα2b for 1 h. In these experiments, IFNα2b-induced p-STAT1 and p-STAT2 did not increase following a second stimulation in WT cells. In contrast, in p.(A219V) cells, phosphorylation of STAT1 and STAT2 was as high as in unprimed cells (Fig. 4b, c), indicating that STAT2 p.(A219V) failed to induce IFNAR2 desensitization. In keeping with this, MX1 and ISG15 expression remained elevated in p.(A219V) cells compared to WT cells, even 36 h after the first IFNα2b stimulation was removed (Fig. 4b, lane 3 and lane 7; 4c). Similarly, cells with the previously described p.(R148W) mutant also showed persistent STAT1 and STAT2 phosphorylation, and elevated MX1 and ISG15 protein expression (Fig. S2b, c), upon a second IFNα2b stimulation.

To explore the basis of this impaired negative feedback response, we investigated the role of USP18. USP18 is a negative regulator in IFN-I signalling, mediating IFNAR2 desensitization in combination with STAT2 [8]. Since USP18, itself an ISG product, was induced in p.(A219V) cells to a higher amount (Fig. 3d, e), we reasoned that it was a defective STAT2-USP18 binding, rather than a lack of USP18, that was responsible for the excessive IFN-I signalling. To investigate this possibility, we performed co-immunoprecipitation in human embryonic kidney (HEK) 293FT cells transiently transfected with STAT2 WT or p.(A219V), together with USP18 (Fig. 4d). These experiments showed a more than 50% decrease in USP18 pulled down by STAT2 p.(A219V) compared to WT (Fig. 4e), indicating defective binding of STAT2 p.(A219V) to USP18.

**Fig. 5:**
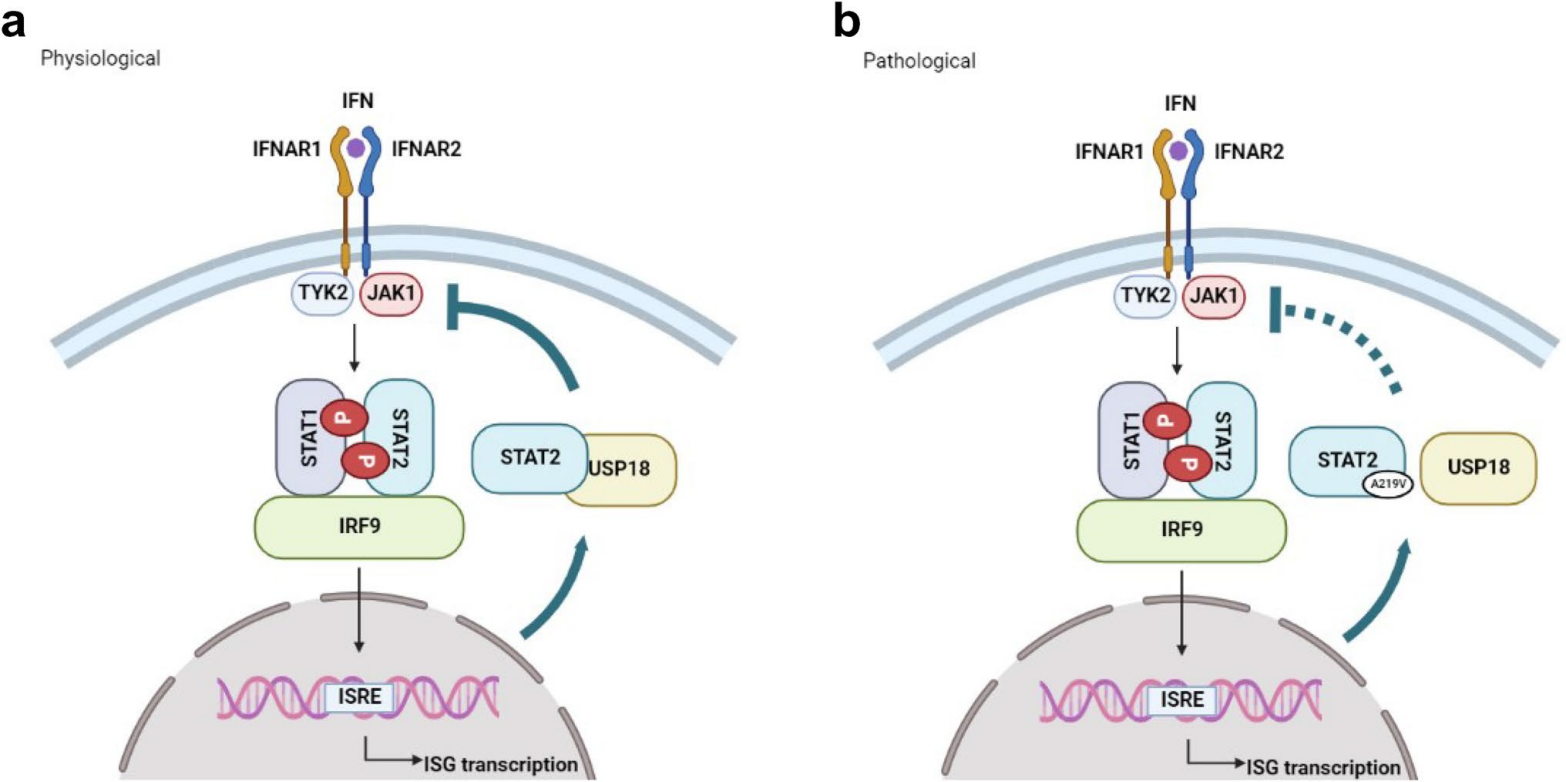
Model of type I interferon (IFN) signalling pathway and regulation. **a** Upon IFN binding, IFNAR1 and IFNAR2 activate TYK2 and JAK1, respectively, via phosphorylation, which in turn phosphorylate STAT1 and STAT2. Phosphorylated STAT1 and STAT2, together with IRF9, form the ISGF3 complex that enters the nucleus to bind genes with an ISRE element, thereby initiating an ISG transcriptional cascade. Under physiological conditions, in the later stages of the IFN response, USP18, can bind STAT2 and together, they displace JAK1 from IFNAR2, thus restricting IFN signalling. **b** When STAT2 loses the ability to bind USP18, e.g., due to STAT2 p.(R148W) or p.(A219V), the negative regulation of IFN signalling is impaired, leading to an enhanced and prolonged IFN response. Graphs were created on BioRender

Collectively, the above data suggest a model in which the homozygous STAT2 p.(A219V) mutation disrupts the interaction of STAT2 with USP18, leading to a failure of negative feedback regulation of IFN-I signalling and prolonged IFNAR activation and enhanced ISG expression (Fig. 5). In silico deep mutagenesis of the predicted STAT2-USP18 interface highlighted a further 3 amino acid residues (E144, D151, and R223) as critical to mediating this interaction (Fig. S3), all clustering in the STAT2 CCD domain in close proximity to the described mutant A219 and R148 residues.

## Discussion

In this study, we report a newly identified homozygous STAT2 mutation in a patient with features of a type I interferonopathy. As an essential protein with dual functions in both transducing and restricting IFN-I signalling, deleterious STAT2 mutations are expected to be rare. Complete STAT2 deficiency can result in a primary immune deficiency and life-threatening viral disease [20]. In contrast, biallelic mutations affecting the negative regulatory function of STAT2 cause a type I interferonopathy state, where patients demonstrate auto-inflammation [9, 10]. To our knowledge, only three patients from two families with homozygous STAT2 mutations have been reported on PubMed to date [9, 10], in both cases involving the R148 residue. Of note, the three patients described in these previous reports all died in infancy. In contrast, the patient in the current study has survived into his third decade. While this observation might relate to a difference in the severity of the mutations involved, our analysis does not support that possibility (see Fig. S2). Alternatively, there may be related genetic and/or environmental issues at play. We note that since only four cases (including ours) have been described to date, it is too early to draw conclusions as to the breadth of phenotype associated with such mutations in STAT2.

The data in this study indicate that mutant STAT2 p.(A219V) retains the ability to transduce IFN-I signalling, yet its negative regulatory function is impaired due to defective USP18 binding, much like the reported p.(R148W) mutation [9]. In contrast, the other reported mutation, p.(R148Q), was described to retain USP18-binding capacity, but the STAT2 p.(R148Q)-USP18 heterodimer could not traffic appropriately to IFNAR2 to displace JAK1 [10]. Despite the described difference in USP18-binding capacity, in both cases, IFN-I signalling was prolonged due to loss of negative IFN-I regulation by STAT2-USP18. R148 and A219 are located within the CCD domain of STAT2, critical to the interaction of STAT2 with USP18 and thus inhibition of IFN-I signalling [8], with in silico deep mutagenesis of the predicted STAT2-USP18 interface highlighting a further 3 amino acid residues (E144, D151, and R223) as potentially critical to this interaction, and in which mutations might be expected to result in prolonged IFN-I signalling due to loss of negative regulation by STAT2-USP18. Of note, defective negative feedback regulation of IFN-I signalling has also been reported in the case of a USP18 mutation, USP18-I60N, resulting in a type I interferonopathy phenotype due to an impaired interaction of USP18-I60N and STAT2 [21].

In their report, Gruber et al. termed the p.(R148Q) mutation as conferring a ‘‘gain-of-function’’ [10]. In our opinion, while an up-regulation of IFN-I signalling is observed, from a molecular perspective, the consequence of the substitution is a loss of a negative regulatory function of STAT2 on IFN-I signalling. Here, we focus on molecular pathology, so that in agreement with the definition set out by Backwell and Marsh [22], and with the International Union of Immunological Societies 2022 update of the phenotypical classification of human inborn errors of immunity [23, 24], we refer to the mutations at R148 and A219 as ‘‘loss-of-function’’.

Another class of mutations observed in STAT2 results in loss of protein expression, with STAT2 deficiency causing a primary immunodeficiency and susceptibility to severe viral diseases [20, 25, 26]. Notably, in a recent report, Gothe et al. [27] described a complete deficiency of STAT2 in patient cells to suppress, but not completely abrogate, IFN-I signalling after IFNα2b stimulation. In this scenario, downstream IFN-I signalling was abnormally prolonged, as evidenced by JAK1 and STAT1 phosphorylation kinetics, in line with a failure of STAT2-USP18-mediated negative regulation. Induction of classical ISGs such as MX1, RSAD2, and IFI44L by IFN-I was suppressed. However, genes with a gamma-activated sequence (GAS) displayed elevated expression, mimicking the IFN-γ effect, which utilized p-STAT1 dimer as a transcriptional activator. These findings were suggested to explain the “paradoxical” observation of autoinflammation in such cases. All of these reports highlight the importance, and complexity, of the regulation of IFN-I signalling by STAT2 [28].

## Supplementary Information

Below is the link to the electronic supplementary material.

Supplementary file1 (PDF 713 KB)
