# Supplementary material for: Type I Interferonopathy due to a Homozygous Loss-of-Inhibitory Function Mutation in STAT2
Source: J Clin Immunol. 2023 Feb 8;43(4):808–18. doi: 10.1007/s10875-023-01445-3 (PMC10110676; doi:10.1007/s10875-023-01445-3)
Supplement: Supplementary file 1 — Supplementary file1 (PDF 713 KB) [file 10875_2023_1445_MOESM1_ESM.pdf]

a

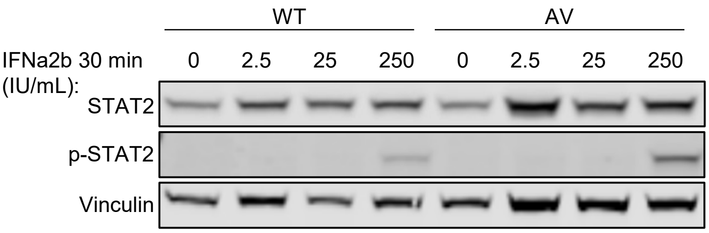

b

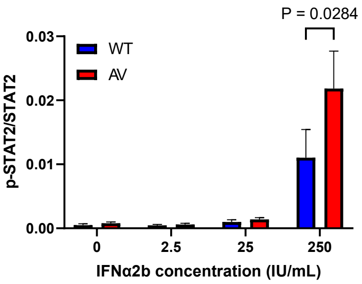

Fig. S1 STAT2 p.(A219V) demonstrates heightened proximal activation upon a short-time IFN stimulation. **a** Immunoblotting analysis of cells stably transduced with STAT2 WT or p.(A219V) (“AV”) and stimulated with 0, 2.5, 25, or 250 IU/mL IFNα2b for 30 min. **b** Densitometry quantification as in **a**. n=3; two-way Anova with Sidak’s multiple comparisons test

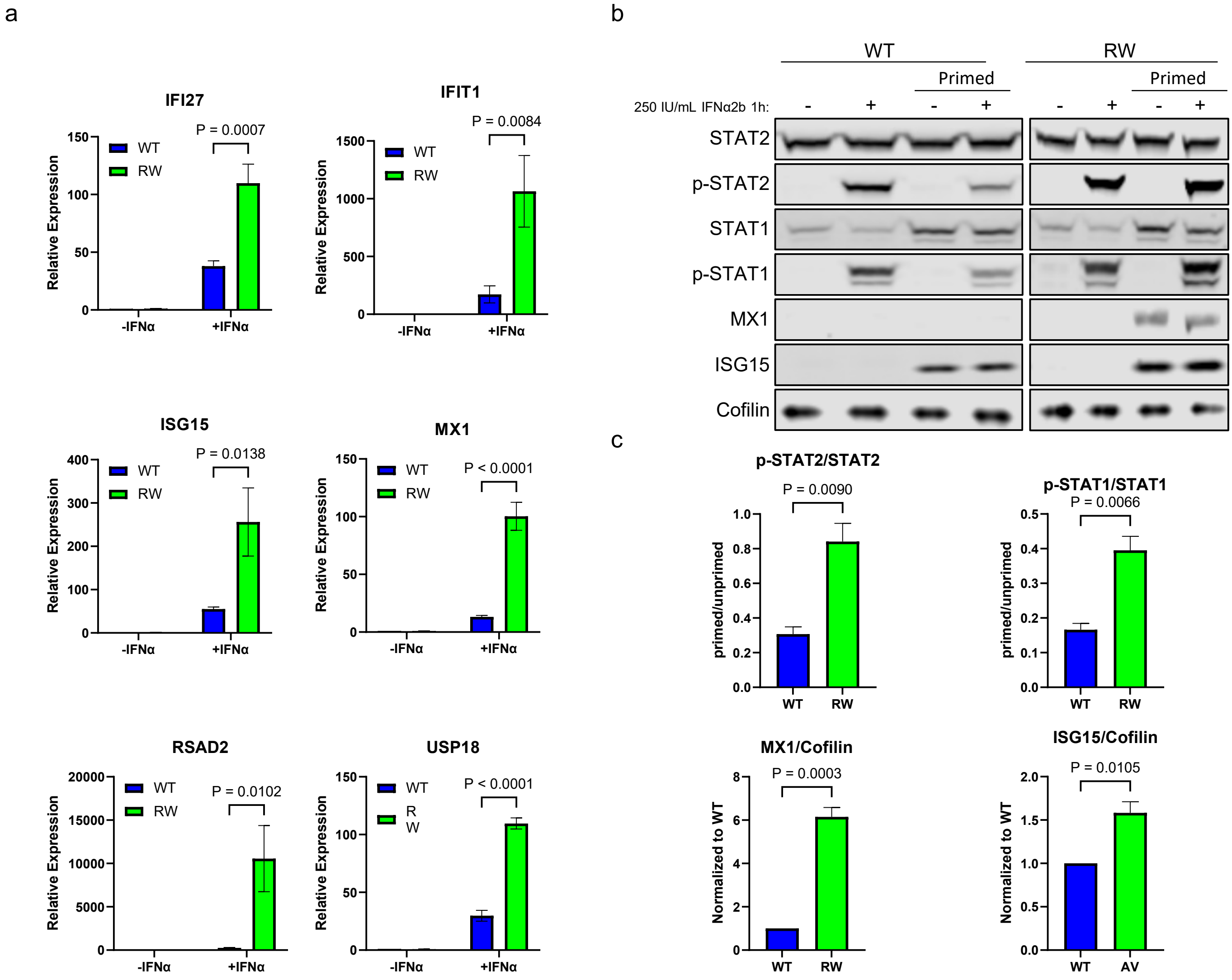

Fig. S2 Enhanced IFN signalling and ISG expression in cells transduced with STAT2 p.(R148W). **a** qPCR analysis of ISG transcription in U6A cells stably transduced with either STAT2 WT or p.(R148W) ('RW') and stimulated for 16 h with 250 IU/mL IFNα2b. n = 3; two-way Anova with Sidak's multiple comparisons test. **b** Immunoblotting analysis to assess the negative regulatory function of STAT2 p.(R148W) by stimulating cells with (''primed''), or without, a priming stimulus. (Blots for p.(R148W) were obtained in the same experiment alongside WT and p.(A219V). Blots for WT shown here are the same blots in **Fig. 4b**.) **c** Densitometry quantification as in **b**. n=3; unpaired *t* test

a

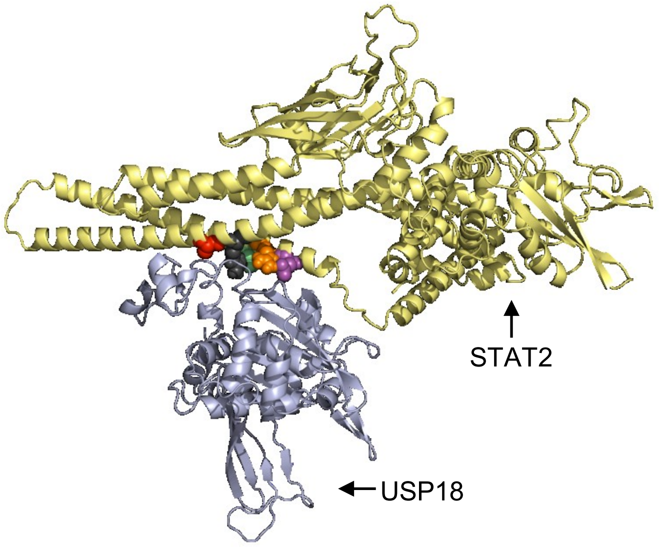

Fig. S3 STAT2-USP18 structural model. **a** STAT2 is depicted in yellow and USP18 in pale blue. The reported amino acid residue arginine 148 (orange) and the alanine 219 (red) in this study are highlighted, along with glutamic acid 144 (purple), aspartic acid 151 (green) and arginine 223 (black), in whom mutations are predicted to disrupt the STAT2-USP18 interaction similar to A219 and R148
